# Agency-preserving robotic assistance for grasp slip recovery in body-powered prostheses

**DOI:** 10.3389/frobt.2025.1675955

**Published:** 2025-10-09

**Authors:** Benjamin Davis, Michael Abbott, Hannah S. Stuart

**Affiliations:** 1 Embodied Dexterity Group, Department of Mechanical Engineering, University of California Berkeley, Berkeley, CA, United States; 2 Design for Assistive Robotic Technologies (DART) Laboratory, Department of Mechanical Engineering, Santa Clara University, Santa Clara, CA, United States

**Keywords:** robotic assistance, prosthetic hand, body-powered, sense of agency, slip reflex

## Abstract

Existing studies demonstrate that performance in reaction-based tasks can be improved using external robotic assistance without reducing the user’s sense of agency, particularly when assistance is delivered near the user’s natural reaction time. This finding has promise for assistive technologies like upper limb prostheses, where agency contributes to long-term use and users’ natural slip reflexes are hindered by reduced feedback and proprioception. However, prior studies lack the physical feedback of device movement inherent to many assistive devices like body-powered prostheses or exoskeletons where user and device are physically coupled. In this work, we explore the relationship between robotic assistance, performance, and agency when such feedback is present. We study how the timing of robotic assistance alters performance and agency, as experienced through the feedback of a body-powered transmission. We collect data from twenty participants in a simulated slip reaction task using a custom body-powered prosthesis emulator, with robotic grasp assistance provided at various delays relative to the onset of slip. Results show that, as assistance becomes more aligned with reaction times, agency increases while performance benefits are still obtained, even if users are aware of the assistance and perceive an increase in performance. Our findings suggest that in scenarios where users can physically perceive robotic assistance and its benefits, such as in body-grounded assistive technologies like body-powered prostheses or exoskeletons, temporal alignment between the user and robotic assistance plays a role in both performance and user experience.

## 1 Introduction

Humans quickly adjust in response to unexpected changes to grasp state during object manipulation. If an object begins to slip out of a grasp, compensatory *local* reflexes can begin in as little as 50 milliseconds (Macefield et al., 1996; Zangrandi et al., 2021). These unconscious reflexes, governed by our nervous system, rapidly adapt to disturbances across scenarios as we interact with a variety of objects in different environments. For prosthesis users, however, reduced sensory feedback, system latency, and unfamiliar control interfaces impede these reflexive actions (Zangrandi et al., 2021). Such users rely heavily on visual feedback and higher level cognitive processes to produce corrective reactions (Sobuh et al., 2014; Thomas et al., 2021). Reliance on visual rather than tactile feedback has been shown to delay response times in reaction time tasks (Chan and Ng, 2012) and increase cognitive loads for prosthesis users (Yamada et al., 2016). Inducing local robotic reflexes that act without conscious intervention from the user presents a potential pathway for reducing cognitive load and speeding up these response times due to faster rates of sensing, processing, and actuation.

Robotic reflexes can react to slip events in robotic manipulation (Nakagawa-Silva et al., 2019; SaLoutos et al., 2022) and improve human performance in other time-critical reaction tasks like simple reaction tasks such as screen tapping or button pressing (Kasahara et al., 2019), balance recovery (Beck et al., 2023; Emmens et al., 2018; Farkhatdinov et al., 2019), and traffic collision avoidance (Wen et al., 2021; Koerten et al., 2024). In the context of prosthetic hands, researchers show how robotic reflexes can improve grasp security by responding to slip events (Engeberg and Meek, 2013; Osborn et al., 2016; Thomas et al., 2023) or predictively adjusting grasp strength (Ray and Engeberg, 2018). These works use myoelectric prostheses, which measure muscle activity with surface electromyography (sEMG) electrodes to control motors that move the end-effector. This use of fully motorized actuation, without body-powered actuation included, allows for integration of new grasp automation strategies without considering physical interactions between the user and device. However, users frequently cite a lack of sensory feedback as a downside of using myoelectric devices (Biddiss et al., 2007). Ongoing research seeks to implement haptic feedback (Stephens-Fripp et al., 2018) or develop new neural integration methods to enhance myoelectric devices (Nghiem et al., 2015), but commercial use remains limited.

People with limb absence often use body-powered prosthetic hands, a passive alternative to myoelectric options (Biddiss and Chau, 2007). Body-powered prehensors rely on movement of the contralateral shoulder to control an end-effector mechanism without motors. This control topology, known as Extended Physiological Proprioception (EPP) (Simpson et al., 1974), provides inherent force and position feedback to the user during operation, reducing the need for artificial haptic feedback. Users of body-powered prostheses complete grasps more quickly and size their gripper apertures more accurately than those of myoelectric prostheses (Gonzalez et al., 2021; Spiers et al., 2021). However, unintended object slip remains a reason for device dissatisfaction across both device categories (Biddiss and Chau, 2007; Kyberd et al., 2007).

We therefore expect that the integration of robotic slip detection and reflexes into body-powered prostheses could improve grasp functionality and user satisfaction while maintaining the benefits of EPP inherent to such devices. Recent work demonstrates body-powered systems equipped with augmentative robotic capabilities, such as a body-powered prehensor with a robotic continuously variable transmission (McPherson et al., 2023). A demonstration of what robotic slip compensation might look like with this device is shown in the supplementary video included with this work.

Prior human factors research reveals that users prefer to remain in control of systems they operate (Shneiderman and Plaisant, 2004). This sense of agency, defined here as a person’s subjective feeling of being responsible for their actions and the associated consequences (Chambon et al., 2014), is often described with the comparator model. This framework states that agency arises from matches between the predicted sensorimotor outcome of an action and the experienced sensory feedback of that action (Blakemore et al., 2001; Chambon et al., 2014). Past work demonstrates how the presence of robotic assistance can reduce the user’s sense of agency over a system by causing misalignment between expected and actual outcomes (Berberian et al., 2012; Berberian, 2019). When the sense of agency is reduced or removed through automation, users tend to lose task motivation (Eitam et al., 2013), take less responsibility for actions (Moretto et al., 2011), and lack preparedness to solve problems that may arise (Berberian, 2019; Koerten et al., 2024). The sense of agency also has specific importance to prosthetic devices, as it contributes to device embodiment, a critical experience for device acceptance (Zbinden et al., 2022; Smail et al., 2021). These findings demonstrate that agency should be considered alongside functional improvements with robotic assistance for effective adoption.

Recent studies demonstrate how user agency can be preserved in the presence of robotic assistance. For example, individuals report an increased sense of agency if robotic assistance improves their performance in controlling a system with unpredictable latency or other disturbances (Wen et al., 2015; Aoyagi et al., 2021). This remains true even if the presence of assistance is made known (Inoue et al., 2017). These disturbances complicate the relationship between action and somatosensory feedback, making continuous comparisons between actions and somatosensory feedback difficult. This results in users relying more heavily on higher level context- and outcome-dependent cues like overall task performance to make perceptual judgments regarding their sense of agency (Moore et al., 2009). However, some researchers observe that this positive effect of performance on agency may not occur in scenarios where users have a strong feeling of control over the unassisted system (Morita et al., 2022). The timing of assistive elements shows promise as another way to modulate the sense of agency, particularly for time-critical reaction tasks. By delaying robotic assistance relative to the onset of some stimulus, individuals demonstrate improved performance in a car braking scenario while reporting comparable agency scores to unassisted conditions (Wen et al., 2021). In a tapping reaction task, Kasahara et al. show that assistance delivered in a time window aligned with normative human reaction times can lead to faster reactions with no significant decrease in agency (Kasahara et al., 2019). These works suggest that the observed preservation of agency during assistance is due to users incorrectly attributing robot-assisted actions to themselves. However, users in these studies do not receive explicit physical feedback regarding the state of the assistive agent, which may limit such misattributions if provided. Furthermore, these studies do not observe trends in agency when assistance is delivered after the user reacts. In imperfect systems where this scenario is possible, the user’s experience may be diminished.

With robotically-assisted body-powered prostheses, EPP enables users to perceive any movements of the device that are not their own, potentially disrupting their sense of agency. For example, changes in the mechanical advantage of a continuous variable transmission body-powered prosthesis (McPherson et al., 2023) could be perceived through changes in force applied to the contralateral shoulder. To develop a better understanding of the effects of robotic intervention in this context, we present the first study on the effect of robotic assistance delay when explicit physical feedback of the robot’s actions is communicated to the user. We hypothesize that, when assistance delays coincide with natural reaction times, users will suppress slip events more quickly while maintaining their sense of agency over the device in accordance with findings from Kasahara et al. (2019). As assistance becomes less temporally aligned with the user (i.e., too early or too late), we predict that the distinction between user and robot actions will become more apparent, and user agency will decrease. We also measure the user’s perceived assistance, perceived task performance, and perceived robot cooperativeness as secondary metrics and compare their trends across assistance conditions with those of agency and performance. We hypothesize that each of these perceptions will contribute to the sense of agency–i.e. that assistance which is less perceivable, more cooperative, and less noticeably beneficial will lead to a higher sense of agency. This is because we expect assistance with these subjective qualities to generate movements more closely aligned with the user’s expectations.

In this work, we present results from a slip reaction task under a variety of robotic assistance conditions using a body-powered prosthesis emulator. Section 2 describes the emulator testbed, robotic assistance, task description and protocol, performance metrics, and methods used for statistical analysis. In Section 3, we present results that highlight the effect of robotic assistance on task performance and the sense of agency. Discussion of these results, experiment limitations, and future work takes place in Section 4. We end with concluding statements in Section 5.

## 2 Materials and methods

### 2.1 Participants

Data represent a total of 20 participants (8 female, 12 male) with an average age of 26.9 (SD: 8.09) and normative upper limb function, i.e., without limb differences or amputation. No participants had past exposure to the system through previous studies. Participants were recruited from the UC Berkeley student population and surrounding communities. All experimental procedures are approved by the University of California, Berkeley Institutional Review Board protocols #2019-05-12178 and #2025-02-18248.

### 2.2 Testbed

**FIGURE 1:**
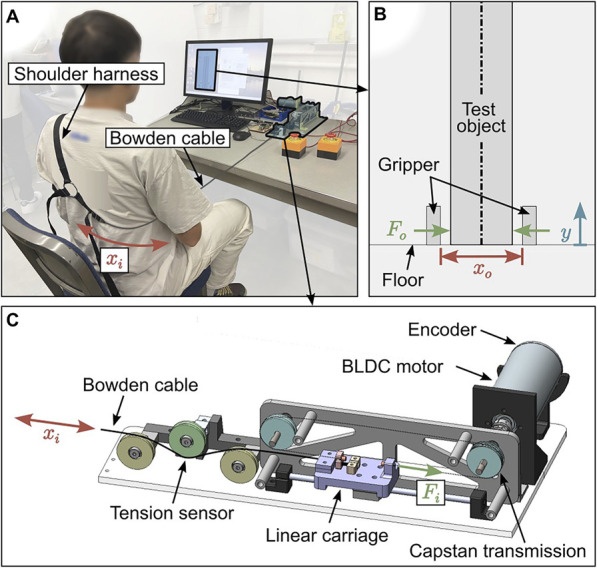
An overview of the prosthesis emulator testbed **(A)** with detailed views of the experimental GUI **(B)** and haptic interface **(C)**. Motion of the user’s contralateral shoulder, $x_{i}$ , determines grasp aperture $x_{o}$ and force $F_{o}$ in the experimental GUI. The haptic interface measures cable excursion and outputs cable force $F_{i}$ back to the user. Adapted from Abbott and Stuart (2023).

To evaluate the effect of robotic assistance on the performance and agency of body-powered prostheses, we adapt the prosthesis emulator described in Abbott et al. (2021) for a virtual slip reaction task. We use an emulator to achieve faster, more efficient execution of repeated, identical trials under varying conditions. Although this limits the generalizability of our results, we aim to establish general trends in delay, performance, and agency through this controlled environment. The testbed, shown in Figure 1, is composed of three subsystems: a figure-of-nine shoulder harness, a desktop haptic interface, and an experimental graphical user interface (GUI) depicting the task in a virtual environment. While the shoulder harness and haptic interface hardware are reused from Abbott et al. (2021), we redesign the haptic interface experimental software, controller, and the GUI to provide a new robot-assisted grasping experience. This redesigned interface enables the addition of a robotic element that can simultaneously affect the virtual gripper state along with the user, and communicates the resulting force feedback to the user.

The impedance-type haptic interface, detailed in Figure 1C, provides participants with positional control of a “right-handed” virtual gripper via motion of their contralateral (left) shoulder and provides force feedback based on grasp forces generated in the virtual environment, mimicking the typical operation of body-powered prostheses. The virtual environment, pictured in Figure 1B, contains two opposing gripper fingers and an infinitely long test object between them which initially rests on a temporary “floor”. The object’s length ensures that it never drops past an unrecoverable state, allowing us to capture all performance data on a continuous scale. We have included a demonstration of the testbed in our supplemental video.

A Bowden cable connects the user’s shoulder harness to the impedance-style haptic interface, which measures positional inputs from the user and outputs force feedback back to the user. We constrain cable motion to one degree of freedom with a linear carriage and translate it into the shaft rotation of a brushless DC (BLDC) motor (Maxon, EC-i 52) using a capstan transmission. An encoder attached to the motor shaft (Maxon, ENC 16 EASY) measures the shaft rotation $x_{i}$ , which is scaled and offset to determine the gripper aperture $x_{o}$ :

$$x_{o}\left(t\right)=x_{o,0}-K_{p}x_{i}\left(t\right)-A\left(t\right)$$

where $x_{o,0}$ is the initial aperture, $K_{p}$ is a position mapping gain chosen to imitate a real body-powered device, and A(t) represents robotic assistance as a function of time. Grasp force on the test object $F_{o}$ is estimated from the gripper aperture and contact stiffness $k_{c}$ , and then multiplied by a force mapping gain $K_{f}=K_{p}^{-1}$ to determine the force to be displayed to the user, $F_{i}=K_{f}F_{o}$ . We use an inline tension sensor and PI controller to deliver this desired force through the cable.

The behavior of the test object in motion is governed by the simple one-dimensional dynamic model

$$\ddot{y}\left(t\right)=\frac{2\mu_{k}F_{o}\left(t\right)}{m}-g,$$

which determines the object’s acceleration $\ddot{y}\left(t\right)$ as a function of the kinetic friction coefficient $\mu_{k}$ , grasp force $F_{o}$ , object mass m , and gravity g . An overview of all object and environment parameters are as follows: mass m=1 kg, width w=60 mm, static and kinetic friction coefficients $\mu_{s}=1$ and $\mu_{k}=0.7$ , contact stiffness $k_{c}=5$ N/mm, gravity g=9.81 m/s^2^, and terminal velocity $V_{t}=500$ mm/s. The terminal velocity limits the amount of force required to quickly stop the test object as it falls. We select these parameters experimentally to produce a reasonably realistic experience while also being easily repeatable without noticeable fatigue.

### 2.3 Robotic assistance

**FIGURE 2:**
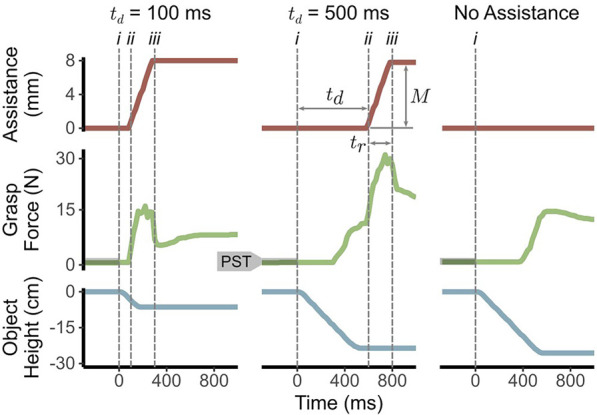
Sample assistance, grasp force, object height data measured over time for an individual trial from two assistance conditions ( $t_{d}=100$ ms, $t_{d}=500$ ms) and the unassisted condition. The delay $\left(t_{d}\right)$ , duration $\left(t_{r}\right)$ , and magnitude (M) of assistance are labeled for the $t_{d}=500$ ms trial. Dashed grey vertical lines mark i) the beginning of object slip, ii) the start of assistance ramp-up, and iii) the end of assistance ramp-up after which it remains constant. Both assistance trials use $t_{r}=200$ ms and M=8 mm. The Pre-Slip Threshold (PST) for grasp force is shown by the shaded grey regions in the grasp force plots.

In this experiment, we introduce robotic assistance as a reflexive closing of the gripper. We set the assistance profile as a linear decrease in aperture added to the user’s positional input commands, shown in the top row of Figure 2. We define the assistance using three parameters, visualized in Figure 2: 1) the *delay* $\left(t_{d}\right)$ after the start of object slip before the automated reflex begins, 2) the *duration* $\left(t_{r}\right)$ of the ramp-up in assistance, and 3) the total *magnitude* (M) reached after the ramp-up. An increase in assistance corresponds to a decrease in gripper aperture. Once assistance reaches M , we maintain that assistance level for the remainder of the trial and reset it before the next trial begins. We express this profile as:

$$A\left(t\right)=\left\{\begin{array}{cc} 0\; & t<t_{d}\\ \frac{M}{t_{r}}\left(t-t_{d}\right)\; & t_{d}\leq t<t_{d}+t_{r}\\ M\; & t\geq t_{d}+t_{r} \end{array}\right.$$

where t is defined as the time relative to the start of object slip. Since user input and assistance are added together, users can partially or fully counteract assistance with their own motion. By defining assistance in this way, we provide participants with a shared control experience representative of interactions with a real robot-assisted body-powered prosthesis (e.g., McPherson et al., 2023). While we reset assistance to 0 between trials for consistency, choosing how and when this reset occurs in practice is not trivial and warrants further investigation. Assistance, grasp force, and object height data from representative trials can be seen in Figure 2.

### 2.4 Experimental procedure

To begin, participants sit facing a screen with the experimental GUI and are fitted with the shoulder harness. The haptic interface is homed and set to a fixed position, and the participants are instructed to reposition their chairs until the Bowden cable reaches light tension with their backs straight and shoulder blades adducted.

#### 2.4.1 Trial protocol

Each trial consists of stopping a falling test object by closing the gripper. Initially, the object rests on a disappearing floor, as seen in Figure 1B. To begin the trial, the participants gently grasp the object with a force within a Pre-Slip Threshold (PST, shown in Figure 2) for a random duration between 0.5 and 1.5 s to limit anticipatory movements. After this threshold is met, the floor disappears and the test object begins falling. The PST is defined as 0.0491 N $<F_{o}<$ 1.96 N, or between 0.007 and 0.28 times the force required to bring the sliding object to a stop (7.01 N). We determined this threshold experimentally to balance difficulty of initiating a trial with consistency in starting grasp force across trials. Participants are instructed to stop the object “as quickly as possible” once slip is observed. After they stop the object and maintain their grasp for at least 1.5 s, the trial ends and the environment is reset. Visual cues are provided in the GUI to guide participants through each trial. We demonstrate this trial procedure in the supplementary video.

#### 2.4.2 Test conditions

To observe the effects of assistance delay $t_{d}$ , we vary its value while holding assistance duration $t_{r}$ and magnitude M constant. In the experiment, we set seven different delay conditions, varying from 0 to 600 m in 100 m intervals, and one unassisted condition for baseline comparisons. We choose this range of delays to observe trends when assistance is delivered before, during, and after the average visual reaction times of 200–300 m observed in pilot testing and literature (Beggs and Howarth, 1970). We opted for 100 m intervals to keep the experiment manageable and limit participant fatigue. For the unassisted condition, we set A(t)=0 and participants are in sole control of gripper position. For all assistance conditions, we define M=8 mm and $t_{r}=200$ ms, which we experimentally determined for subject comfort and task effectiveness. If there is no positional input from the user $\left(x_{i}=0\right)$ , the provided assistance is sufficient to stop the object on its own.

#### 2.4.3 Experimental structure

Before collecting trial data, participants perform a warm-up of five unassisted trials and five trials with immediate assistance $\left(t_{d}=0\right)$ . We inform them that the two conditions demonstrate the lowest and highest levels of assistance to be experienced, respectively, and that all experimental conditions will provide a level of assistance at or between these levels. During data collection, participants experience the eight conditions in pseudorandomly ordered blocks. Each condition block involves an unrestricted number of practice trials, 20 experimental trials, and then a set of survey questions relating to their experience. Participants indicate to the experimenter when they are ready to move from practice trials to data-collection trials. Open-ended questions are asked at the end of each condition block and after the entire experiment to provide general sentiments on the different conditions.

### 2.5 Metrics

#### 2.5.1 Task performance metrics

Force, gripper position, and object height data are recorded at a rate of 50 Hz for each experimental trial. Data from practice trials are not included in our analysis. Since the assistance delay conditions are in intervals of 100 m and expected reaction times are on the order of hundreds of milliseconds, we expect this rate to be sufficient for observing our trends of interest. The object is considered stopped once its height remains unchanged for 400 m after its initial slip. This duration is less than the required 1.5 s hold in the task, as we noted some participants would accidentally release the object early. We assess trial performance by measuring the object slip distance. We also estimate reaction times for each participant as the time required to reach an incremental increase in grasp force greater than 1 N after initial object slip under the unassisted condition. While reaction time is not a direct performance metric, we use it to contextualize results from our discrete assistance timing conditions.

#### 2.5.2 Survey metrics

After each condition, participants answer a set of survey questions (see Table 1). Four seven-point Likert questions, adapted from previously published work on agency (Bekrater-Bodmann, 2020; Kalckert and Ehrsson, 2012), evaluate participant sense of agency. These questions are positively or negatively worded regarding the sense of agency and scored accordingly. The scores are summed to obtain an aggregate agency score between four and 28. We also measure the perceived assistance provided by each delay condition on a ten-point scale. We include two more seven-point Likert questions to measure each participant’s perceived improvement in stopping the falling object relative to the unassisted condition (*Improved Performance*) and the perceived cooperation of the robotic assistance in completing the task (*Cooperated*). We include these questions on perceived assistance, improvement, and robot cooperation, listed in Table 1, to explore what factors might contribute to the sense of agency.

**TABLE 1:** Post-condition survey questions.

Measure	Question	Scale
Agency	The gripper was moving in the way I wanted it to move	7-point Likert
	I was in control of the gripper	7-point Likert
	The gripper seemed to move on its own	7-point Likert
	I was responsible for stopping the object	7-point Likert
Performance	The robotic grasp assistance improved my performance in stopping the object quickly	7-point Likert
Cooperation	The robotic grasp assistance helped me complete the task rather than attempting to complete the task independently	7-point Likert
Assistance	Relative to the minimum and maximum assistance conditions experienced at the beginning of the experiment, rate the assistance magnitude for this condition	10-point numeric

### 2.6 Statistical analysis

A mixed model regression evaluates the effects of assistance delay on each metric. We treat assistance delay as the primary fixed effect, with condition order (CO) and trial number (TN) as covariates to account for potential learning or fatigue effects. We consider delay as an ordinal variable to enable pairwise comparisons between conditions. The unassisted condition is treated as infinitely delayed assistance and ordered accordingly. We use polynomial contrasts to fit a seventh-order polynomial to these eight ordinal conditions, but present only up to quartic trends (.L, .Q, .C, and 0.4̂) for simplicity. To achieve model parsimony for each dependent variable, we use likelihood ratio tests to identify significant autoregressive effects and interaction effects. These comparisons are detailed in the Supplementary Material *.*

To analyze slip distance results, we use a linear mixed model and a first-order autoregressive correlation structure for observed autocorrelation of lag 1. We use cumulative link mixed models for agency, perceived performance, perceived robot cooperation, and perceived magnitude of assistance measures, as recommended in Taylor et al. (2023) for ordinal data. For all models, participant ID is added as a random effect to account for within-subject correlation.

Post-hoc pairwise comparisons for all dependent variables, except perceived robot cooperation, use the Tukey’s Honest Significant Difference test, which corrects for family-wise error rate. Comparisons are made between the model-based estimated marginal means for each assistance delay condition. We focus only on pairwise comparisons of assistance conditions to the unassisted condition in this work with the aim of evaluating the effect of assistance on performance and agency. Since the perceived robot cooperation question assumes that robotic assistance was present, answers from the unassisted condition are not considered and no pairwise comparisons are made. Regression coefficients reveal trends in all metrics for different assistance delay values. Model outputs can be found as Supplementary Material *.*

To further investigate the effect of assistance delay on agency, we analyze the relationship between agency and the timing of assistance relative to median subject reaction times $\left(t_{d}-t_{\mathrm{reaction}}\right)$ using repeated measures correlations to account for within-subject dependence. We run separate correlations to determine trends in agency before and after subject reaction times. These correlations only include agency results from assisted conditions, as $t_{d}$ is undefined for the unassisted condition.

All statistical analyses are performed with R v4.3.1. Linear mixed modeling for slip distance is achieved with the “nlme” package (Pinheiro et al., 2023), and cumulative link mixed modeling for survey metrics is achieved with the “ordinal” package (Christensen, 2023). Estimated marginal means are calculated with the “emmeans” package (Lenth, 2024). The repeated measures correlations are accomplished with the “rmcorr” package (Linden, 2021).

Due to the complexity of our models, we performed a Monte Carlo power analysis with preliminary data to estimate the required sample size. We selected two pairwise comparisons to assess, with effect sizes pulled from the preliminary data: (1) we evaluated agency scores between the 300 m and 400 m delay conditions, assuming an odds ratio of 9.6 in favor of the 400 m condition, and (2) we compared drop distance between the 400 m and unassisted conditions, assuming an effect size of 6.4 cm. Synthetic data was sampled based on variances observed in initial data and analyzed with the same model structures used for our main analysis, i.e., a linear mixed model for drop distance and a cumulative link mixed model for agency. For agency, the 300 m–400 m pairwise comparison was selected as our primary comparison of interest, as we expected reaction times to be aligned with one of these conditions more than the other and thus show a slightly higher agency score based on our hypothesis. We selected the 400 m - unassisted pairwise comparison for drop distance, since we expected the 400 m condition to be the most delayed assistance that still provides a performance benefit. With 10,000 replications, both pairwise conditions showed > 80% power with 20 subjects for α=0.05 . We did not perform power analyses on secondary metrics like perceived assistance, perceived performance, and perceived cooperation, as these metrics were more exploratory in nature.

## 3 Results

### 3.1 Task performance

**FIGURE 3:**
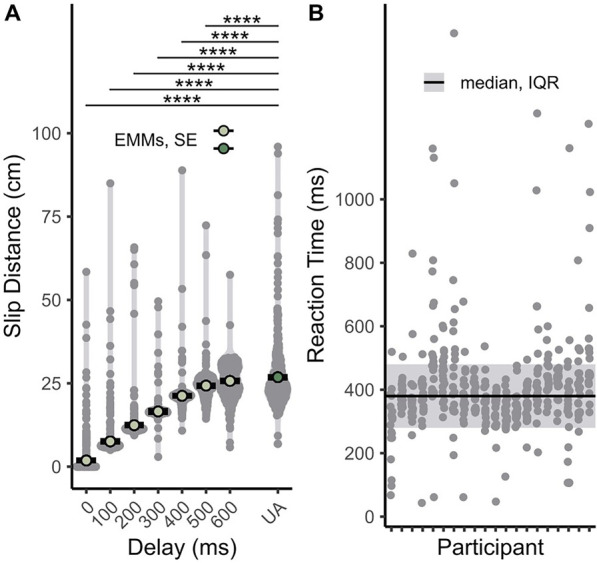
Summary of results from slip distance across different assistance delays **(A)** and participant reaction times **(B)**. In **(A)**, smaller grey dots represent raw data points, and larger colored dots represent estimated marginal means. Black horizontal bars depict standard error. The unassisted condition is represented by the “UA” label and dark green dot. Asterisks denote significant pairwise comparisons between estimated marginal means of the assisted and the unassisted conditions (* =p<0.05 , ** =p<0.01 , *** =p<0.001 , **** =p<0.0001 ). The solid horizontal line and shaded region in **(B)** depict the overall median and IQR, respectively.

As expected, longer assistance delay generally results in a larger object slip distance, as shown in Figure 3A. Participants exhibit a positive linear effect ( $t_{d}$ .L: b=21.4 , p<0.0001 ), negative quadratic effect ( $t_{d}$ .Q: b=−7.18 , p<0.0001 ), negative cubic effect ( $t_{d}$ .C: b=−3.07 , p<0.0001 ), and negative quartic effect ( $t_{d}$ .^4: b=−2.18 , p=0.00884 ) for slip distance with an increasing delay of assistance. The positive linear effect shows that slip distance generally increases with delay. The significant negative quadratic, cubic, and quartic effects imply that the overall slope decreases at the tail end of conditions, meaning the positive linear effect diminishes at longer delays. This is likely because at longer delays, the user can react with or before the robot, limiting the performance benefits from assistance. Slip distance also increases with condition order (CO.L: b=0.123 *,* p=0.0211 ), suggesting that fatigue occurs as the experiment goes on. Pairwise tests reveal that assistance provided at 500 m or earlier improves performance, improving slip recovery relative to the unassisted condition by between 2.53 cm (500 m delay) and 25.0 cm (0 m delay) on average.

In the unassisted trials, we estimate the median reaction time to be 380 m (interquartile range, IQR: 100 m) across all participants, shown in Figure 3B. This average is slightly longer than laboratory-recorded reaction times to visual stimuli of 200–300 m (Beggs and Howarth, 1970; Keele and Posner, 1968). We suspect that reaction times are slower due to an unfamiliar shoulder-driven mode of control for users and a larger and slower joint in the shoulder than the hand or fingers typically used in reaction time tests.

In the performance metrics, several visual outliers show abnormally low slip distance (Figure 3A) and fast reaction times (Figure 3B). We attribute these results to participants anticipating the onset of slip. Visual outliers with unusually high slip distances also represent cases of poor performance. Long reaction times in Figure 3B are likely due to loss of concentration. However, large slip distances in Figure 3A indicate that the proposed method of assistance does not consistently improve task performance. This outcome, possibly due to inconsistent human input, is further explored in Section 4.

### 3.2 Subject survey

**FIGURE 4:**
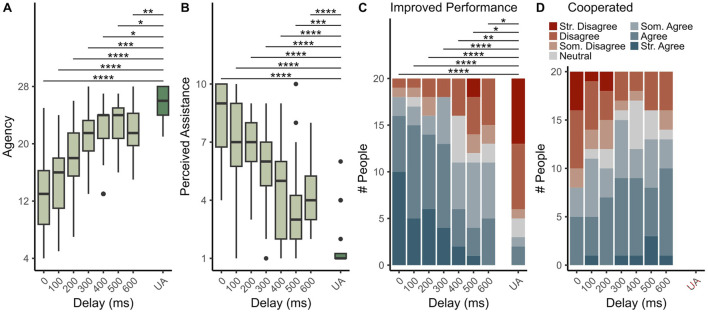
Summary of survey results regarding agency **(A)**, perceived assistance **(B)**, perceived performance **(C)**, and perceived robot cooperation **(D)**. The unassisted condition is represented by the “UA” label and dark green boxes in **(A)** and **(B)**. Asterisks denote significant pairwise comparisons between estimated marginal means of the assisted and the unassisted conditions (* =p<0.05 , ** =p<0.01 , *** =p<0.001 , **** =p<0.0001 ). In **(D)**, unassisted data and associated comparisons are not shown since the question assumes the presence of assistance.

As shown in Figure 4A, increases in assistance delay generally improve participants’ sense of agency, with an observed positive linear effect of delay on agency ( $t_{d}$ .L: b=5.03, p<0.0001 ). However, this increase in agency appears to be nonlinear, as evidenced by a positive quartic effect ( $t_{d}$ .^4: b=1.25 , p=0.00218 ) of delay on agency. This trend indicates that agency may reach a local maximum at some intermediate delay. Although pairwise comparisons between the unassisted condition and assisted conditions show that no assistance conditions allow users to totally maintain their sense of agency, they also support the existence of a local maximum, with assistance at 400 or 500 m delays showing higher senses of agency than other delay conditions on average, though direct comparison tests are left for future work.

Figure 4B shows that subjects perceive less assistance at longer delays ( $t_{d}$ .L: b=−6.06, p<0.0001 ), and a significant negative quartic effect is also present ( $t_{d}$ ^4: b=−1.18, p=0.00576 ). Subjects appear to be aware of the robotic assistance regardless of delay time, as revealed by significant comparisons between the unassisted condition and all assisted conditions. However, the quartic trend and pairwise comparisons indicate that a local minimum may be present, with the 500 m condition being perceived as lowest across all assistance conditions on average. This trend mirrors what the data shows for agency, indicating that perceptions of assistance and agency may be negatively correlated.

Subjects perceive a decrease in performance with longer assistance delays ( $t_{d}$ .L: b=−4.12, p<0.0001 ), as shown in Figure 4C. Pairwise tests reveal that all assistance conditions improve perception of performance compared to the unassisted condition, even with a 600 m delay where no actual performance benefit occurs. Therefore, the presence of robotic assistance alone appears to positively impact user perceptions of task performance. Notably, subjects who perceive no assistance (i.e., perceived assistance = 1) for any assisted condition also disagree with seeing an improvement in performance in those conditions.

Results in Figure 4D show that robotic assistance feels more cooperative to subjects as its delay increases ( $t_{d}$ .L: b=1.33,p=0.00210 ). The 300–500 m delay conditions appear to generate the strongest feelings of cooperation with the fewest negative responses, but this result is supported by only marginal significance of the negative quadratic effect of delay ( $t_{d}$ .Q: b=−0.755, p=0.0753 ). Since the unassisted condition is not considered, no pairwise comparisons are made. Thus, this result primarily suggests a possible increase in perceived robot cooperation with increasing delay, with some suggestion that this trend may plateau or reverse at long delays.

**FIGURE 5:**
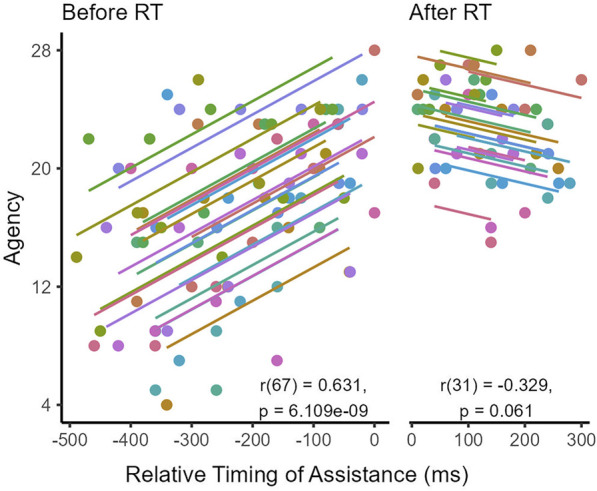
Repeated measures correlations between agency and assistance timing relative to each subject’s median reaction time (RT), done separately for before and after RT data. Annotations list degrees of freedom, strength, and significance for each correlation.

Figure 5 shows a significant, moderate positive correlation between agency and relative assistance timing when assistance is delivered before each subject’s average reaction time (r(67)=0.631, p<0.0001) . The trend reverses for assistance delivered after subjects begin to react, with correlation results revealing a marginally significant negative trend (r(31)=−0.329, p=0.0610) . These results indicate that temporal synchrony between the user and robot is positively linked with the sense of agency.

### 3.3 Statistical interactions

We observed significant interactions with condition order for slip distance (CO.L: b = 0.497,p = 0.000943 , CO.Q: b = 0.478,p = 0.00337 , CO.C: b = 0.505,p = 0.00149 , CO.4̂: b = 0.467,p = 0.0.00401 ). These interaction coefficients, while minor relative to the fixed effects coefficients, increase the linear effects and reduce the quadratic, cubic, and quartic effects of delay on slip distance over time. While the exact cause for these interactions is unclear, we suspect that subject learning and fatigue may play a role. In the case of learning, subjects may see an improvement in task performance and their ability to discern between delay conditions. Additionally, fatigue may contribute to the increasing linearity of trends as subjects become slower and more reliant on assistance to stop the object. For the survey metrics, models without interactions between delay and condition order were selected for better model parsimony via Akaike Information Criterion (AIC) score (Akaike, 1998), so no interaction effects are presented.

## 4 Discussion

Our performance and agency results show that robotically improving slip reactions with our emulator reduces the user’s sense of agency. These findings reaffirm that robotic assistance reduces user agency in shared control systems (Berberian et al., 2012; Berberian, 2019) and expand this idea into a physical human-robot interaction scenario. However, our results also show that this tradeoff is dependent on the relative timing of assistance. We find that agency increases as assistance is delayed to align with user reaction times (i.e., $t_{d}$ = 400 or 500 m), agreeing with earlier findings (Kasahara et al., 2019). We additionally show that agency appears to decrease if assistance is delayed beyond user reaction times, indicating that assistance alignment with user reaction times may have unique benefits for the user experience. Our results imply that assistance aligned with user reaction time may improve functional outcomes with minimal reduction in user metrics like embodiment, satisfaction, and motivation, even if the presence of assistance is evident to the user through haptic feedback.

A few subjects report high agency scores while failing to identify the presence of assistance when it aligns with their reaction times, perhaps mistaking robotic movements as their own. This phenomenon is hypothesized in prior work where physical feedback of assistance is not apparent (Kasahara et al., 2019; Inoue et al., 2017; Morita et al., 2022). In these works, authors propose that when feedback of task outcomes aligns closely enough with the user’s expectations, robot-assisted actions can be self-attributed. In our study, results indicate that the presence of assistance is largely recognized, likely due to the physical feedback of assistance delivered to the user. Still, subjects exhibit high levels of agency, particularly with delays of 400–500 m. Assistance in these conditions, although noticeable, is described by subjects as “natural” and “synchronous” and perceived by a majority as cooperative. These comments and results suggest that the retention of agency is not primarily due to the misattribution of gripper movement. Instead, we propose that force feedback makes the robotic assistance explicit, leading users to recognize the robot as a co-actor capable of contributing to the task’s outcome. In this case, users might derive their sense of agency from their perceptions of the robot’s intentions (Navare et al., 2024). Another explanation for high levels of agency even when assistance is noticeable is that users may be embodying the device as a tool (Gibson, 2014; Maravita and Iriki, 2004). In this case, the user may understand that the robotic reflex enhances the device’s existing grasp affordance and learn how to utilize it appropriately. However, this incorporation of the device into the user’s own body schema requires expertise, which may or may not be obtained during our 2 hour experiment (Weser and Proffitt, 2021). In either case, based on our results, we propose that the retention of agency during robotic assistance is still possible, provided the robot acts synchronously and predictably.

The results in Figure 3A show that even when performance is improved, the object can still fall large distances ( > 20 cm) before being stopped. These distances may be amplified due to the unfamiliarity of the shoulder-actuated gripper and the use of an emulator, which does not provide feedback on object weight and momentum. With these limitations in mind, we focus primarily on the trends in our data rather than the practical outcomes. In practice, we expect subject reaction times to be closer to those published in literature (Beggs and Howarth, 1970; Keele and Posner, 1968), which would result in shorter slip distances allowed. In this case, we predict that assistance provided at shorter delays (e.g., 200–300 m) would generate the highest average sense of agency for users across the assistance conditions.

Visual outliers present in the performance data (Figure 3A) show that the proposed assistance to body-powered grasping does not always suppress slip fully. We suspect that these results are due to the shared control scheme of a robot-assisted body-powered prosthesis. Since users are always mechanically coupled to the end-effector, they can counteract the provided assistance through their own movements and decrease the system’s overall effectiveness. Thus, consistent improved performance requires some level of cooperation, and therefore knowledge of the system, from the user to avoid inadvertently diminishing the effects of assistance.

Perceived performance improvements through assistance at any delay may lead to positive outcomes like increased motivation (Almagro et al., 2020) and confidence (O’Brien et al., 2001), even when assistance has not improved actual performance (i.e., $t_{d}$ = 600 m). However, unlike findings from previous work (Wen et al., 2015; Inoue et al., 2017; Aoyagi et al., 2021), improvements in perceived or actual performance do not improve the sense of agency in this work. In the mentioned studies, subjects likely focused on external cues like task performance to establish agency (Moore et al., 2009), as action-feedback comparisons were complicated by a lack of haptic feedback and disturbance of control schemes (e.g., input latency). On the other hand, body-powered prostheses provide users with straightforward control and inherent feedback, allowing for clear action-feedback comparisons to establish the sense of agency that require less reliance on external cues. Similar to findings in Morita et al. (2022), we find that a strong sense of agency with the unassisted device prevents agency from improving with increased performance.

Though assistance aligned with measured reaction times could improve slip suppression and maintain the user’s sense of agency, subjects express that other timings may also have benefits. For assistance provided with little or no delay ( $t_{d}\leq 200$ ms), some participants note its reduction of necessary user effort (e.g., task was “much easier”), although others report dissatisfaction with the lack of control. The most delayed assistance ( $t_{d}=600$ ms) received positive comments, with multiple subjects reporting a feeling of control and that the assistance responds to their movements to provide support (e.g., the robot “reacts [to their motion] to stop the object” and provides a “secure hold”). Taken collectively, these results suggest user satisfaction of robotic assistance may be more nuanced depending on individual user preferences. Future work will examine how users might prefer different balances of performance and agency based on task goals or device expectations.

### 4.1 Limitations and future work

This study is performed with a sample population of twenty individuals with a relatively low average age and without limb difference. While this population of naive users can provide insight on the experience of individuals new to limb loss, it prevents us from understanding the experiences of long-term prosthesis users and different age populations less familiar with robotic systems. Future studies with an increased number of participants from a broader population, as well as the inclusion of prosthesis users, is necessary to generalize the results of this work.

Our experimental design balances experimental resolution and breadth, which leads to some limitations. Our choice of delay conditions prevents us from using methodologies that make precise conclusions on optimal assistance timing, such as that proposed by Kasahara et al. (2019), and capturing events where assistance is much later than user reaction times. The range and granularity of delays could be expanded to obtain more detailed and comprehensive trends in all metrics. Furthermore, subjects interact with only one set of object properties (e.g., mass, size, and stiffness) for an unbreakable object and one set of assistance magnitude (M) and ramp time $\left(t_{r}\right)$ values. The lack of object breakability or any other encouraged grasp force limits means that overgrasp may occur without penalty. Overgrasp is an important outcome to consider when assistance is added onto user movements like in our setup and should be investigated in future work. Different assistance parameters could impact the effectiveness or perceptibility of assistance, and user preferences in assistance behavior may change for different object types. Future work should explore alternative assistance designs, as well as how factors like user expertise and device instructions impact the effectiveness of assistance across various tasks (e.g., Zhou et al., 2021).

The testbed used in this work isolates the force and proprioceptive feedback of body-powered prosthesis operation. Yet, the virtual environment does not provide sensations of weight or momentum of the test object, which has been shown to affect reaction times and compensation strategies (Häger-Ross and Johansson, 1996). The mechatronic system also introduces more latency and exhibits lower precision than the purely mechanical connection present in body-powered devices. Future work will seek to implement robotic slip reflexes in physical robot-augmented body-powered devices (e.g., McPherson et al. (2023); Mcpherson et al. (2020)) and evaluate how trends in performance and agency are impacted by use of a physical device.

While our findings are promising for the incorporation of physically perceptible assistance in wearable devices, we acknowledge that the form of feedback subjects receive in our study is unique to body-powered prostheses. Translation of our results to other devices with different paths of control and feedback requires future study.

## 5 Conclusion

In this work, we explore the effect of automated assistance delay on task performance and user agency using a desktop body-powered prosthesis emulator with a robotic slip reflex. We find that as robotic assistance is provided closer to subject reaction times, perceived agency increases and performance benefits are still obtained. This occurs even though subjects typically recognize the presence of assistance, likely due to the force feedback present in the system, and perceive an improvement in performance. Qualitative results show that subjects feel “in sync” with the assistance when provided at these delays. Our findings support unique considerations for future design of robotic assistance, particularly for body-grounded assistive technologies like body-powered prostheses and exoskeletons where robotic movements cannot be masked. In these situations, we propose reaction time-aligned assistance as a promising way to improve reflex-based performance with minimal reduction in user agency.

## Data Availability

The raw data supporting the conclusions of this article will be made available by the authors, without undue reservation.
