# Supplementary material for: Agency-preserving robotic assistance for grasp slip recovery in body-powered prostheses
Source: Front Robot AI. 2025 Oct 9;12:1675955. doi: 10.3389/frobt.2025.1675955 (PMC12545140; doi:10.3389/frobt.2025.1675955)
Supplement: Supplementary file 1 [file DataSheet1.pdf]

Supplementary Material

1 STATISTICS FOR MIXED MODELS AND PAIRWISE COMPARISONS

1.1 Drop Distance

Model Term	Estimate	Std. Error	t-value	p-value
t_d.L	21.36	0.73	29.22	2.10e-166
t_d.Q	-7.18	0.84	-8.50	2.83e-17
t_d.C	-3.07	0.77	-3.99	6.67e-5
t_d.^4	-2.18	0.83	-2.62	8.84e-3
CO	0.12	0.05	2.31	0.02
TN	-3.15e-3	0.02	-0.18	0.86
t_d.L:CO	0.50	0.15	3.31	9.43e-4
t_d.Q:CO	0.48	0.16	2.93	3.37e-3
t_d.C:CO	0.50	0.16	3.18	1.49e-3
t_d.^4:CO	0.47	0.16	2.88	4.01e-3

Table S1. Drop distance model coefficients up to the quartic polynomial contrast. The notation .L, .Q, .C, and .^4 represent the linear, quadratic, cubic, and quartic effects, respectively.

Contrast	Estimate	Std. Error	t-value	p-value
0 - UA	-24.98	0.47	-53.20	0
100 - UA	-19.27	0.46	-41.59	0
200 - UA	-14.40	0.46	-31.03	0
300 - UA	-10.28	0.54	-19.14	0
400 - UA	-5.59	0.46	-12.08	0
500 - UA	-2.53	0.47	-5.38	2.18e-6
600 - UA	-1.12	0.46	-2.41	0.23

Table S2. Drop distance pairwise comparisons on estimated marginal means.

Compared Models	df	AIC	Log Lik.	p-value	R_c^2	R_m^2
DD $\sim (t_d + \text{CO} + \text{TN})^2$ + (1|ID)	28	20285.68	-10114.84	-	0.701	0.682
DD $\sim (t_d + \text{CO} + \text{TN})^2$	27	20491.83	-10218.92	<0.0001	-	-
DD $\sim (t_d + \text{CO} + \text{TN})^2$ + (1|ID)	28	20285.68	-10114.84	-	0.701	0.682
DD $\sim (t_d + \text{CO} + \text{TN})^2 - t_d$:TN + (1|ID)	21	20280.91	-10119.46	0.2365	0.700	0.681
DD $\sim (t_d + \text{CO} + \text{TN})^2$ + (1|ID)	28	20285.68	-10114.84	-	0.701	0.682
DD $\sim (t_d + \text{CO} + \text{TN})^2 - t_d$:CO + (1|ID)	21	20315.16	-10136.58	<0.0001	0.696	0.676
DD $\sim (t_d + \text{CO} + \text{TN})^2$ + (1|ID)	28	20285.68	-10114.84	-	0.701	0.682
DD $\sim (t_d + \text{CO} + \text{TN})^2 -$ CO:TN + (1|ID)	27	20284.92	-10115.46	0.2663	0.701	0.682
Final Model	DD $\sim t_d$ + CO + TN + t_d:CO + (1|ID)					

Table S3. Model selection comparisons for drop distance (DD) are shown by two consecutive rows, where row lines separate different comparisons. We list the compared models, degrees of freedom (df), Akaike Information Criterion (AIC), log likelihood, and conditional and marginal R^2 values R_c^2 and R_m^2. We also include the p-value of rejecting the null hypothesis that the simpler model is sufficient. We use shorthand notation for the models, where (t_d + CO + TN)^2 includes the main effects t_d (assistance delay), CO (condition order), and TN (trial number), and the interaction effects t_d:CO, t_d:TN, and CO:TN. The notation "(t_d + CO + TN)$^2-$CO:TN" implies the interaction B:C has been removed from the model. The notation (1|ID) represents the inclusion of participant ID as a random intercept. The second model in the first comparison does not have a random effect, so R_c^2 and R_m^2 cannot be calculated. The final row shows the selected model.

1.2 Agency

Contrast	Estimate	Std. Error	z-value	p-value
t_d.L	5.03	0.55	9.22	2.96e-20
t_d.Q	-0.77	0.44	-1.75	0.08
t_d.C	0.64	0.41	1.57	0.12
t_d.^4	1.25	0.41	3.06	2.18e-3
CO	-0.03	0.06	-0.43	0.67

Table S4. Agency model coefficients up to the quartic polynomial contrast. The notation .L, .Q, .C, and .^4 represent the linear, quadratic, cubic, and quartic effects, respectively.

Contrast	Estimate	Std. Error	z-value	p-value
0 - UA	-6.13	0.73	-8.37	8.50e-14
100 - UA	-5.23	0.68	-7.69	4.53e-13
200 - UA	-4.05	0.64	-6.30	8.41e-9
300 - UA	-2.64	0.63	-4.22	6.53e-4
400 - UA	-1.92	0.59	-3.23	0.03
500 - UA	-1.83	0.59	-3.09	0.04
600 - UA	-2.25	0.61	-3.72	4.94e-3

Table S5. Agency pairwise comparisons on estimated marginal means.

**Compared Models**	**df**	**AIC**	**Log Lik.**	**p-value**	R_c^2	R_m^2
A $\sim (t_d + \text{CO})^2$ + (1|ID)	38	892.41	-408.20	-	0.567	0.492
A $\sim (t_d + \text{CO})^2$	37	898.99	-412.49	0.0034	-	-
A $\sim (t_d + \text{CO})^2$ + (1|ID)	38	892.41	-408.20	-	0.567	0.492
A $\sim t_d$ + CO + (1|ID)	31	882.09	-410.05	0.815	0.555	0.477
final model	A $\sim t_d$ + CO + (1|ID)					

Table S6. Model selection comparisons for agency (A) are shown by two consecutive rows, where row lines separate different comparisons. We list the compared models, degrees of freedom (df), Akaike Information Criterion (AIC), log likelihood, and conditional and marginal R^2 values R_c^2 and R_m^2. We also include the p-value of rejecting the null hypothesis that the simpler model is sufficient. We use shorthand notation for the models, where $(t_d$ + CO$)^2$ includes the main effects t_d (assistance delay) and CO (condition order) and their interaction effect t_d:CO. The notation (1|ID) represents the inclusion of participant ID as a random intercept. The second model in the first comparison does not have a random effect, so R_c^2 and R_m^2 cannot be calculated. The final row shows the selected model.

1.3 Perceived Assistance

**Contrast**	**Estimate**	**Std. Error**	**z-value**	**p-value**
t_d.L	-6.06	0.65	-9.38	6.85e-21
t_d.Q	-0.60	0.48	-1.25	0.21
t_d.C	-0.81	0.45	-1.80	0.07
t_d.^4	-1.18	0.43	-2.76	5.76e-3
CO	-4.64e-3	0.07	-0.07	0.94

Table S7. Perceived assistance model coefficients up to the quartic polynomial contrast. The notation .L, .Q, .C, and .^4 represent the linear, quadratic, cubic, and quartic effects, respectively.

**Contrast**	**Estimate**	**Std. Error**	**z-value**	**p-value**
0 - UA	7.57	0.90	8.45	7.99e-14
100 - UA	6.67	0.85	7.81	2.08e-13
200 - UA	6.03	0.81	7.40	3.81e-12
300 - UA	5.00	0.79	6.35	6.14e-9
400 - UA	3.70	0.76	4.90	2.65e-5
500 - UA	3.11	0.75	4.17	7.82e-4
600 - UA	3.58	0.75	4.75	5.68e-5

Table S8. Perceived assistance pairwise comparisons on estimated marginal means.

Compared Models	df	AIC	Log Lik.	p-value	R^2_c	R^2_m
PA $\sim (t_d + \text{CO})^2$ + (1|ID)	25	629.76	-289.88	-	0.715	0.554
PA $\sim (t_d + \text{CO})^2$	24	657.65	-304.83	<0.0001	-	-
PA $\sim (t_d + \text{CO})^2$ + (1|ID)	25	629.76	-289.88	-	0.715	0.554
PA $\sim t_d$ + CO + (1|ID)	18	628.42	-296.21	0.0808	0.673	0.506
final model	PA $\sim t_d$ + CO + (1|ID)					

Table S9. Model selection comparisons for perceived assistance (PA) are shown by two consecutive rows, where row lines separate different comparisons. We list the compared models, degrees of freedom (df), Akaike Information Criterion (AIC), log likelihood, and conditional and marginal R^2 values R^2_c and R^2_m. We also include the p-value of rejecting the null hypothesis that the simpler model is sufficient. We use shorthand notation for the models, where $(t_d$ + CO$)^2$ includes the main effects t_d (assistance delay) and CO (condition order) and their interaction effect t_d:CO. The notation (1|ID) represents the inclusion of participant ID as a random intercept. The second model in the first comparison does not have a random effect, so R^2_c and R^2_m cannot be calculated. The final row shows the selected model.

1.4 Perceived Performance

Contrast	Estimate	Std. Error	z-value	p-value
t_d.L	-4.12	0.53	-7.73	1.11e-14
t_d.Q	-0.70	0.46	-1.53	0.13
t_d.C	-0.45	0.43	-1.04	0.30
t_d.^4	-0.30	0.41	-0.72	0.47
CO	-0.03	0.07	-0.38	0.70

Table S10. Perceived performance model coefficients up to the quartic polynomial contrast. The notation .L, .Q, .C, and .^4 represent the linear, quadratic, cubic, and quartic effects, respectively.

Contrast	Estimate	Std. Error	z-value	p-value
0 - UA	5.13	0.75	6.79	3.10e-10
100 - UA	4.21	0.69	6.10	3.04e-8
200 - UA	4.13	0.70	5.87	1.24e-7
300 - UA	3.97	0.69	5.75	2.54e-7
400 - UA	2.58	0.64	4.01	1.54e-3
500 - UA	2.07	0.64	3.23	0.03
600 - UA	2.22	0.63	3.52	0.01

Table S11. Perceived performance pairwise comparisons on estimated marginal means.

Compared Models	df	AIC	Log Lik.	p-value	R^2_c	R^2_m
PP $\sim (t_d + \text{CO})^2$ + (1|ID)	22	521.05	-238.53	-	0.523	0.442
PP $\sim (t_d + \text{CO})^2$	21	525.78	-241.89	0.0095	-	-
PP $\sim (t_d + \text{CO})^2$ + (1|ID)	22	521.05	-238.53	-	0.523	0.442
PP $\sim t_d$ + CO + (1|ID)	15	520.12	-245.06	0.0706	0.473	0.380
final model	PP $\sim t_d$ + CO + (1|ID)					

Table S12. Model selection comparisons for perceived performance (PP) are shown by two consecutive rows, where row lines separate different comparisons. We list the compared models, degrees of freedom (df), Akaike Information Criterion (AIC), log likelihood, and conditional and marginal R^2 values R^2_c and R^2_m. We also include the p-value of rejecting the null hypothesis that the simpler model is sufficient. We use shorthand notation for the models, where $(t_d$ + CO$)^2$ includes the main effects t_d (assistance delay) and CO (condition order) and their interaction effect t_d:CO. The notation (1|ID) represents the inclusion of participant ID as a random intercept. The second model in the first comparison does not have a random effect, so R^2_c and R^2_m cannot be calculated. The final row shows the selected model.

1.5 Perceived Cooperation

Contrast	Estimate	Std. Error	z-value	p-value
t_d.L	1.33	0.43	3.08	2.10e-3
t_d.Q	-0.76	0.42	-1.78	0.08
t_d.C	0.07	0.41	0.17	0.87
t_d.^4	0.05	0.41	0.12	0.90
CO	-0.06	0.07	-0.90	0.37

Table S13. Perceived cooperation model coefficients up to the quartic polynomial contrast. The notation .L, .Q, .C, and .^4 represent the linear, quadratic, cubic, and quartic effects, respectively.

Compared Models	df	AIC	Log Lik.	p-value	R^2_c	R^2_m
PC $\sim (t_d + \text{CO})^2$ + (1|ID)	20	503.56	-231.78	-	0.179	0.141
PC $\sim (t_d + \text{CO})^2$	19	502.19	-232.09	0.4298	-	-
PC $\sim (t_d + \text{CO})^2$ + (1|ID)	20	503.56	-231.78	-	0.179	0.141
PC $\sim t_d$ + CO + (1|ID)	15	498.77	-235.38	0.3023	0.137	0.094
final model	PC $\sim t_d$ + CO + (1|ID)					

Table S14. Model selection comparisons for perceived cooperation (PC) are shown by two consecutive rows, where row lines separate different comparisons. We list the compared models, degrees of freedom (df), Akaike Information Criterion (AIC), log likelihood, and conditional and marginal R^2 values R^2_c and R^2_m. We also include the p-value of rejecting the null hypothesis that the simpler model is sufficient. We use shorthand notation for the models, where $(t_d$ + CO$)^2$ includes the main effects t_d (assistance delay) and CO (condition order) and their interaction effect t_d:CO. The notation (1|ID) represents the inclusion of participant ID as a random intercept. The second model in the first comparison does not have a random effect, so R^2_c and R^2_m cannot be calculated. Even though the random effect did not show significant model improvement, we include it due to study design. The final row shows the selected model.
